# The Predictive Potentiality of Salivary Microbiome for the Recurrence of Early Childhood Caries

**DOI:** 10.3389/fcimb.2018.00423

**Published:** 2018-12-14

**Authors:** Ce Zhu, Chao Yuan, Shuang Ao, Xiangru Shi, Feng Chen, Xiangyu Sun, Shuguo Zheng

**Affiliations:** ^1^Department of Preventive Dentistry, Peking University School and Hospital of Stomatology, National Engineering Laboratory for Digital and Material Technology of Stomatology, Beijing Key Laboratory of Digital Stomatology, Beijing, China; ^2^Central Laboratory, National Engineering Laboratory for Digital and Material Technology of Stomatology, Beijing Key Laboratory of Digital Stomatology, Peking University School and Hospital of Stomatology, Beijing, China

**Keywords:** early childhood caries, recurrence, salivary microbiome, sequencing analysis, predictive potentiality

## Abstract

The aim of this study was to investigate the variation of the salivary microbiota in the recurrence of early childhood caries (ECC), and to explore and verify the potential microbial indicators of ECC recurrence. Saliva samples from kindergarten children were tracked every 6 months for 1 year. Finally, in total 28 children and 84 samples were placed on the analysis phase: 7 children with ECC recurrence made up the ECC-recurrence (ER) group, 6 children without ECC recurrence constituted the non-ECC-recurrence (NER) group, and 15 children who kept ECC-free were set as the ECC-free (EF) group. DNA amplicons of the V3-V4 hypervariable region of the bacterial *16S rDNA* were generated and sequencing was performed using Illumina MiSeq PE250 platform. No statistically significant differences of the Shannon indices were found in both cross-sectional and longitudinal comparisons. Furthermore, both principal coordinates analysis (PCoA) and heatmap plots demonstrated that the salivary microbial community structure might have potentiality to predict ECC recurrence at an early phase. The relative abundance of *Fusobacterium, Prevotella, Leptotrichia*, and *Capnocytophaga* differed significantly between the ER and NER groups at baseline. The values of area under the curve (AUC) of the four genera and their combined synthesis in the prediction for ECC recurrence were 0.857, 0.833, 0.786, 0.833, and 0.952, respectively. The relative abundance of *Fusobacterium, Prevotella, Leptotrichia*, and *Capnocytophaga* and their combination showed satisfactory accuracy in the prediction for ECC recurrence, indicating that salivary microbiome had predictive potentiality for recurrence of this disease. These findings might facilitate more effective strategy to be taken in the management of the recurrence of ECC.

## Introduction

Early childhood caries (ECC) is referred as “the presence of one or more decayed (non-cavitated or cavitated lesions), missing (due to caries), or filled tooth surfaces” in any primary tooth in a child aged 71 months or younger (Drury et al., 1999; Selwitz et al., 2007). ECC is the most common chronic childhood disease (Bagramian et al., 2009) which affects 23% of preschool children in the United States (Dye et al., 2015) and over 60% of children in China (Hu et al., 2011). This disease is a critical public health concern because its characteristics of wide-ranging, rapid-progressing, severe, and difficult to treat. ECC could produce kinds of adverse effects on children both physically and psychologically, posing great economic burden on families and the society (Cummins, 2013).

The current recommendation of care for ECC includes restoration and extraction of carious teeth, topical application of fluoride, and oral hygiene instructions. The incidence of ECC was significantly higher in children with a previous history of the disease (Javed et al., 2017). It was reported that a significant proportion (24–52%) of children treated for ECC will develop new caries lesions within 6 months after the initial treatment (Graves et al., 2004; Amin et al., 2010; Berkowitz et al., 2011). Thus, the management after treatment and prediction of ECC recurrence are of particular clinical significance. Traditional predictors for caries relapse after dental treatment included socio-economic status, general health status, parental compliance of dietary, and oral hygiene instructions, follow-up and recall appointment intervals, and exposure to topical or systemic fluoride. However, these traditional predictors remain controversial (Berkowitz et al., 2011; Amin et al., 2015) with certain limitations (Tellez et al., 2013). Novel approaches based on best evidence are needed to improve the prevention of ECC recurrence.

Currently, classifying and predicting host states based on human microbiota has become one key goal of human microbiome projects worldwide (Knights et al., 2011; Human Microbiome Project Consortium, 2012). Researchers have increasingly agreed with the hypothesis from an ecological perspective to explain the mechanism of caries occurrence and development (Marsh, 2006, 2010; Yang et al., 2012; Jiang et al., 2014), and syntrophic and competitive interactions dominate in oral ecology (Horner-Devine et al., 2007; Lamont et al., 2018). Under this condition, next generation sequencing technology (NGS) provides an important technical means for the study of oral microbial community (Siqueira et al., 2012), and has been used for caries prediction conforming to the ecological plaque hypothesis. As a representative sample of oral ecology, saliva is a non-invasive source containing complex genetic information of human and oral microbes (Baum et al., 2011; Zhang et al., 2016) used extensively in the early diagnosis of dental caries (Vukosavljevic et al., 2011), and its potential value in caries prediction was recently reported (Teng et al., 2015).

Although several recent studies focused on the variation of the oral microbiota during the course of ECC onset or progression to build the biomarker methods in preventing ECC, little is known about ECC recurrence at present: what variation happens on the oral microbiota during the course of ECC recurrence or whether ECC recurrence reflects a particular infection that might be detected at the initial phase. The answer would be helpful for taking appropriate preventive measures at an appropriate time, as well as monitoring and predicting the development of ECC recurrence after the initial treatment.

Thus, our research group conducted a longitudinal study investigating the salivary proteomic profiles in the development of ECC on preschool children aged 3 years old in one kindergarten in Beijing, China (Ao et al., 2017). We utilized NGS to investigate the variation of the salivary microbiota during the course of ECC recurrence, whilst to explore the potential microbial indicators of ECC recurrence, so as to explore if certain microbiota at the post-treatment baseline could predict which children were more likely to develop recurrent ECC, which would potentially facilitate improvements in the strategy for ECC prevention.

## Materials and Methods

### Ethics Approval and Informed Consent

This study was ethically approved by Peking University School and Hospital of Stomatology Ethics Committee (Issuing number: PKUSSIRB-2013060). Parents of the participating children have all signed informed consent prior to enrolment.

### Subjects and Design

This study conformed to STROBE guidelines (Table S1). The sample in this study was a convenience sample from a previous study also conducted by our research group (Ao et al., 2017). In total 82 ECC-free preschool children aged 3 years old in one kindergarten in Beijing were recruited in May 2014 and September 2014. Our previous work was to investigate differences of salivary protein profiles associated with development of caries on 30 selected children after 12 months follow-up periods (Ao et al., 2017). The same 30 children from previous study were selected into the present study, including 15 children diagnosed as ECC and 15 children (who were age- and sex-matched with the ECC children) who had no experience of ECC.

Children meeting more than one of the following criteria were excluded in current study: (1) Ingestion of antibiotics or probiotics within 4 weeks before sampling; (2) Failure to comply with treatment and follow-up requirements; (3) Voluntary withdrawal. The ECC children were completely treated by dentists from the department of Preventive Dentistry, Peking University School, and Hospital of Stomatology. The sampling baseline in this study was at the time point of 2 weeks right after the last clinical measurements. Then these children were followed up every 6 months for up to 12 months. The three time-points were described as time-point 1 (T1, baseline), time-point 2 (T2, 6 months), time-point 3 (T3, 12 months) in turn. At the time point of 6 months after baseline, two of the 15 children with ECC were excluded due to noncompliance. At the end of 12-month follow-up period (T3), we grouped the children by status of ECC recurrence as follows: 7 of the 13 ECC-treated children who had developed new caries lesions were put into ECC-recurrence group (ER); 6 of the 13 children who had no new caries lesions became members of non-ECC-recurrence group (NER); and other 15 children (who were age- and sex-matched with the ECC-treated children) were selected from those who stayed ECC-free and set as control (ECC-free group, EF).

### Saliva Sampling and Total Genomic DNA Sample Preparation

The subjects were instructed to rinse their mouth thoroughly with water before sampling and then rest for at least 10 min before saliva collection at 8:30 a.m. 1.5 mL of stimulated whole saliva samples were collected into a graduated tube through a funnel and immediately placed on ice. Saliva samples were then transferred to the laboratory on ice and stored in a −80°C refrigerator. The supernatant was removed by centrifugation at 10,000 × g for 10 min at 4°C and precipitate were stored at −80°C prior to DNA extraction. Total genomic DNA extraction was performed using QIAamp DNA mini kit (Qiagen, Hilden, Germany) according to manufacturer's instructions. Concentration and purity testing of the DNA was performed using a NanoDrop 8000 spectrophotometer (NanoDrop Technologies, Wilmington, DE, USA). The integrity of bacterial genomic DNA was checked by 1% agarose gel electrophoresis and a negative control only with buffer was used. The DNA samples were stored at −80°C until further use.

### PCR Amplification and Sequencing

Universal primers for *16S rDNA* double V3-V4 region were used to conduct PCR amplification (338F 5′-GTACTCCTACGGGAGGCAGCA-3′, 806R 5′-GTGGACTACHVGGGTWTCTAAT-3′). The cycling conditions were carried out as follows: initial denaturation at 94°C for 5 min, 30 cycles consisting of denaturation at 95°C for 30 s, annealing at 56°C for 30 s, elongation at 72°C for 40 s, and final extension at 72°C for 10 min. A negative control only with buffer was enrolled during DNA amplification to eliminate interference. The quality of the amplified PCR products was detected by 2% agarose gel electrophoresis. The purification of PCR products was performed using a QIAquick Gel Extraction Kit (QIAGEN, Hilden, Germany). The purified PCR products were quantified using a Real-time PCR system. Illumina MiSeq 250 bp paired-end (PE) sequencing platform (Illumina, San Diego, CA, USA) was used for amplified products according to standard operating protocols.

### OTU Profile Analysis

The original data obtained by MiSeq sequencing were processed via Mothur (version 1.35.0) (Schloss et al., 2009) and QIIME (version 1.9.0) (Caporaso et al., 2010). High quality sequences were obtained by removing raw reads shorter than 200 bp in length, with a quality value <20, with mononucleotide repeats and homopolymers >10 bp. High-quality sequences were clustered into Operational Taxonomic Units (OTUs) at 3% dissimilarity (Edgar, 2010) with QIIME software. The normalization was conducted by extraction to balance randomly in each sample to reduce the effects of variable sequencing depths. The bioinformatics analyses of saliva samples were based on the results of the normalized OTU table, and different taxonomic levels (phylum, class, order, family, genus) were classified based on the RDP Classifier (version 2.12, Center for Microbial Ecology, Michigan State University, East Lansing, MI, USA) with the default 0.7 confidence threshold. The UniFrac distance metric was generated based on the OTU phylogenetic tree and OTU relative abundance. Principal coordinate analysis (PCoA) was performed according to the UniFrac distance using a weighted algorithm (Lozupone et al., 2006, 2007).

### Statistical Analysis

The differences in microbial diversity index were analyzed using Kruskal–Wallis test via SPSS version 23.0 (IBM, Armonk, NY, USA), whilst multiple comparison between the groups was performed using all pairwise. The comparisons of the similarities between different groups and different time-points were conducted using Analysis of similarities (Anosim) based on the UniFrac distance. The differentially abundant features at different taxonomic levels between two groups were performed using metastats analysis via Mothur software (Pat Schloss, Michigan, USA). Receiver-operating characteristic (ROC) analysis was conducted using SPSS version 23.0 (IBM, Armonk, NY, USA). *P* < 0.05 was considered as statistically significant and all *P*-values were two-sided.

## Results

### Demographic and Caries Status

A total of 28 preschool children were enrolled in this study at the end of follow-up period (Table 1). Subjects with ECC treatment history in the 1-year duration were divided into the ECC-recurrence (ER) and the non-ECC-recurrence (NER) groups according to the results of dental examination at the time point of 6 months after baseline. The participants' caries status during the 12-month follow-up period are presented in Table 1. The mean age (months) and standard deviation at baseline for preschool children in ER, NER and EF groups were 54.7 ± 4.54, 58.7 ± 4.24, 55.5 ± 6.66, respectively. Meanwhile, the gender proportion (Female/Male) for preschool children in ER, NER and EF groups were 4/3, 2/4, 8/7, respectively. There were no significant differences in the mean age and gender proportion among the three groups (*P* = 0.436, *P* = 0.932, respectively).

**Table 1:** The participants' caries status (decayed teeth) at the three time-points.

**Group**	**Host ID**	**ECC treatment history**	**ds**		
			**T1**	**T2**	**T3**
ER	10	Y	0	2	3
ER	79	Y	0	3	3
ER	88	Y	0	4	6
ER	109	Y	0	1	2
ER	113	Y	0	3	3
ER	114	Y	0	2	4
ER	124	Y	0	2	4
NER	11	Y	0	0	0
NER	14	Y	0	0	0
NER	23	Y	0	0	0
NER	27	Y	0	0	0
NER	28	Y	0	0	0
NER	77	Y	0	0	0
EF	4	N	0	0	0
EF	5	N	0	0	0
EF	15	N	0	0	0
EF	17	N	0	0	0
EF	20	N	0	0	0
EF	21	N	0	0	0
EF	22	N	0	0	0
EF	76	N	0	0	0
EF	78	N	0	0	0
EF	84	N	0	0	0
EF	85	N	0	0	0
EF	89	N	0	0	0
EF	97	N	0	0	0
EF	102	N	0	0	0
EF	111	N	0	0	0

*ER, the ECC-recurrence group; NER, the non-ECC-recurrence group; EF, ECC-free group; ds, the number of decayed tooth surface; T1, for baseline; T2, for the time point of 6 months; T3, for the time point of 12 months; Y, for yes; N, for no*.

### The Diversity of Salivary Microbiota

A total of 84 saliva samples were collected. The sequencing method generated a total of 3,650,544 sequences after quality filtering, with an average of 43,459 (ranged from 18,111 to 86,758) sequences per sample. The normalization was conducted by random extraction to balance in each sample to 18,111. The average read length was 426 bp.

The *16S rRNA* gene reads were then assigned to OTUs at 3% dissimilarity. A total of 222 OTUs were found in the T1, with 207 OTUs in the T2 and 208 OTUs in the T3. The number of shared and unique OTUs in each group and time-point was shown in Figure S1A.

The species richness of the salivary microbiota of each sample was estimated by rarefaction analysis (Figure S1B). The shape of the rarefaction curves evidenced that a plateau was completely reached, indicating that the sequencing depth of all the samples was reasonable and the sequencing results could reflect microbial information in the samples. We constructed specaccum curves for each time-point and for all OTUs detected to assess the current state of sampling (Figure S1C). The specaccum curve in three time-points all reached saturation point at 10 sample-set, indicating that the sampling in EF group (15 samples) was sufficient from the sequencing aspect, whereas the sampling in ER group (7 samples) and NER group (6 samples) might be insufficient from the sequencing aspect.

### The Variation of Salivary Microbiome During the Follow-Up Period

By analyzing the salivary microbiota of all samples, a total of 13 phyla, 22 classes, 38 orders, 50 families, and 85 genera were detected. The overall structure of the salivary microbiota at phylum and genus levels for each group and time-point was shown in Figures S2–S4. The legends exhibited the top20 taxa according to the mean relative abundance.

**Figure 1:**
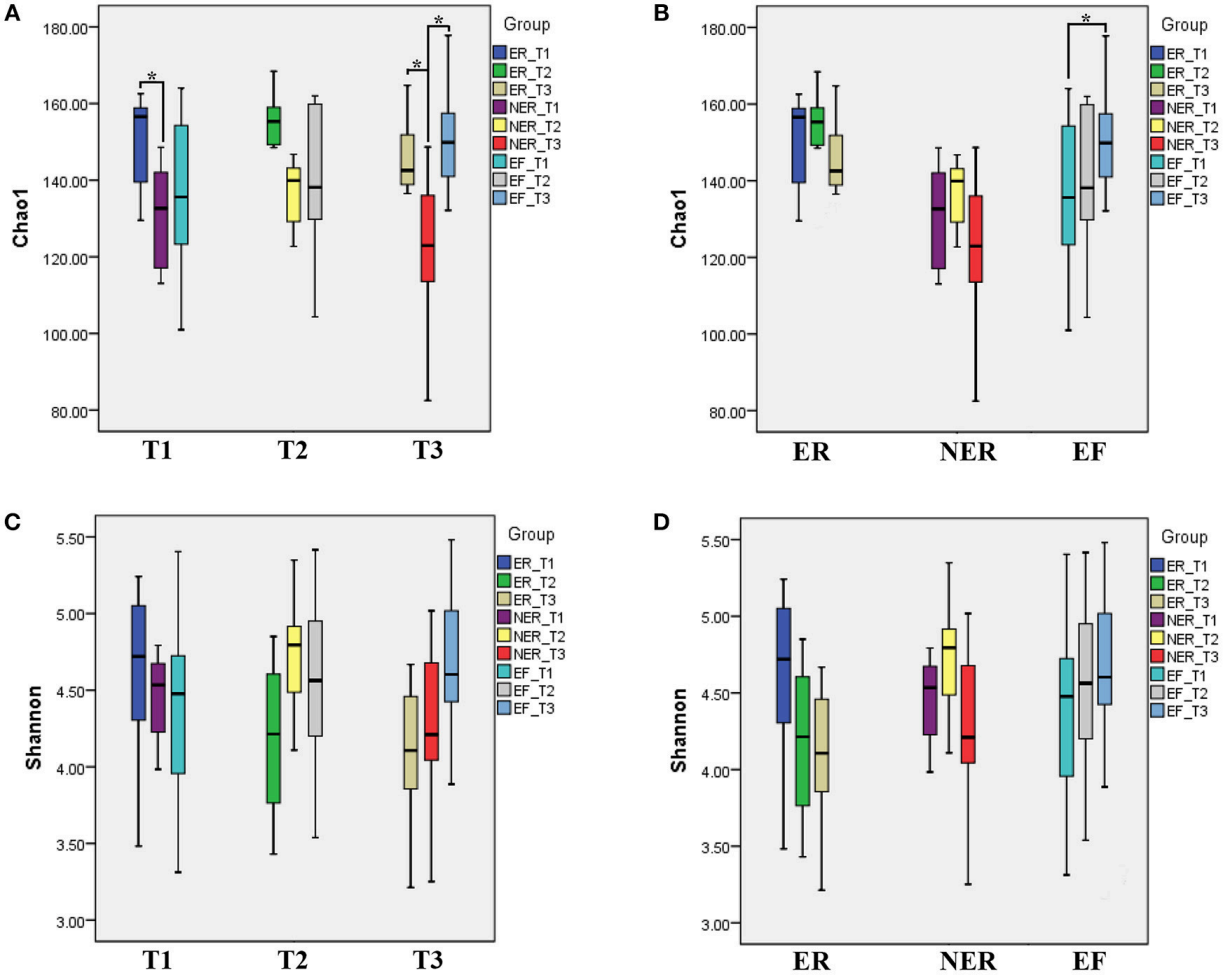
**(A)** The comparison of Chao1 index among three groups within same time point. *P < 0.05. **(B)** The comparison of Chao1 index among three time points within same group. *P < 0.05. **(C)** The comparison of Shannon index among three groups within same time point. **(D)** The comparison of Shannon index among three time points within same group.

The α-diversity indices in each group, including Chao 1 index, observed species index, PD_whole_tree index and Shannon index, were displayed in Table S2. Chao1 and Shannon indices are the most commonly used indices to estimate α-diversity of the microbes. For cross-sectional comparisons of Chao1 index (Figure 1A), we found the Chao1 index in the ER group was significantly higher than that in the NER group at T1 (*P* = 0.035), and the Chao1 index in the NER group was significantly lower than that in the ER and EF groups at T3 (*P* = 0.029, *P* = 0.003, respectively), whereas there were no significant differences amongst all the other comparisons (*P* > 0.05). For longitudinal comparisons of Chao1 indices (Figure 1B), we found the Chao1 index at T1 was significantly lower than T3 in the EF group (*P* = 0.045), whereas there were no significant differences amongst all the other comparisons (*P* > 0.05). By cross-sectional and longitudinal comparisons of Shannon indices (Figures 1C,D), no statistically significant differences were found (*P* > 0.05). However, we found that the Chao1 and Shannon indices in the EF group were rising progressively, indicating that the α-diversity of salivary microbiota was growing with age. It was also found that the Chao1 and Shannon indices in the ER group were decreasing gradually, indicating that the α-diversity of salivary microbiota exhibited a declining trend during the course of recurrence and progression of ECC.

**Figure 2:**
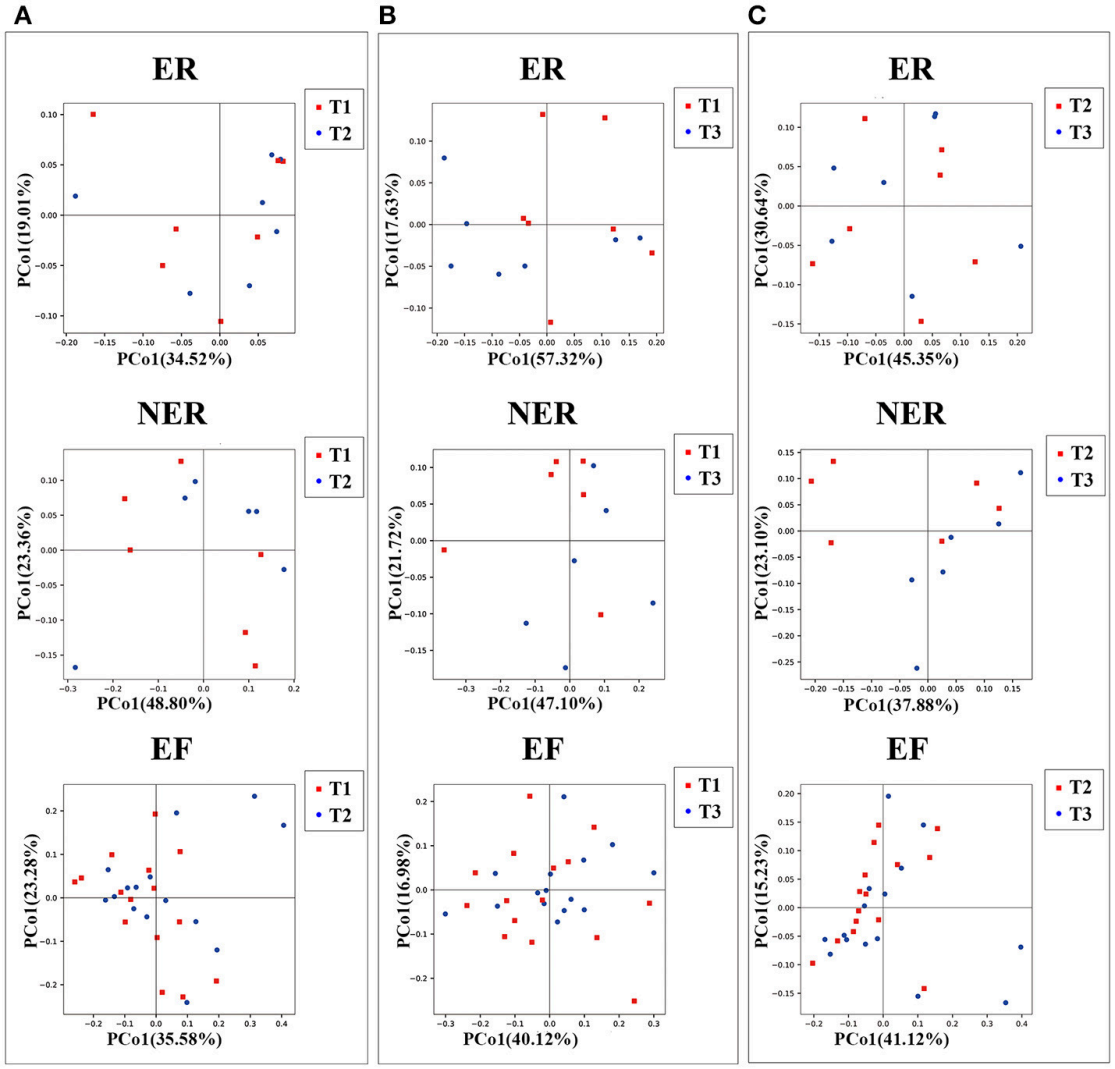
**(A)** Principal coordinates analysis (PCoA) based on weighted UniFrac distance exhibiting the variation of microbiota community structure from T1 to T2 in the three groups. Each symbol represents one sample: red for T1, blue for T2. **(B)** PCoA based on weighted UniFrac distance exhibiting the variation of community structure from T1 to T3 in the three groups. Each symbol represents one sample: red for T1, blue for T3. **(C)** PCoA based on weighted UniFrac distance exhibiting the variation of community structure from T2 to T3 in the three groups. Each symbol represents one sample: red for T2, blue for T3.

β-diversity shown by principal coordinates analysis (PCoA) of weighted UniFrac distance were analyzed to demonstrate the in-group variation of salivary microbial community structure during the follow-up period (Figure 2). The closer the distance between samples shown in the diagram, the more similar the microbial community structure were. The results showed that the salivary microbial community structure did not change significantly from T1 to T2 (Figure 2A, Anosim method, *P* = 0.581 for the ER group, *P* = 0.154 for the NER group, and *P* = 0.486 for the EF group, respectively). No significant change was observed from T2 to T3 as well (Figure 2B, Anosim method, *P* = 0.581 for the ER group, *P* = 0.154 for the NER group, and *P* = 0.486 for the EF group, respectively). The same results were shown in the comparison between T1 and T3 (Figure 2C, Anosim method, *P* = 0.080 for the ER group, *P* = 0.648 for the NER group, and *P* = 0.311 for the EF group, respectively). The results of α- and β-diversity comparisons (shown in Figures 1, 2) were consistent, indicating that the samples in the present study all exhibited similar microbial community structure.

### The Predictive Potentiality of Salivary Microbial Community Structure for the Recurrence of ECC at Baseline

**Figure 3:**
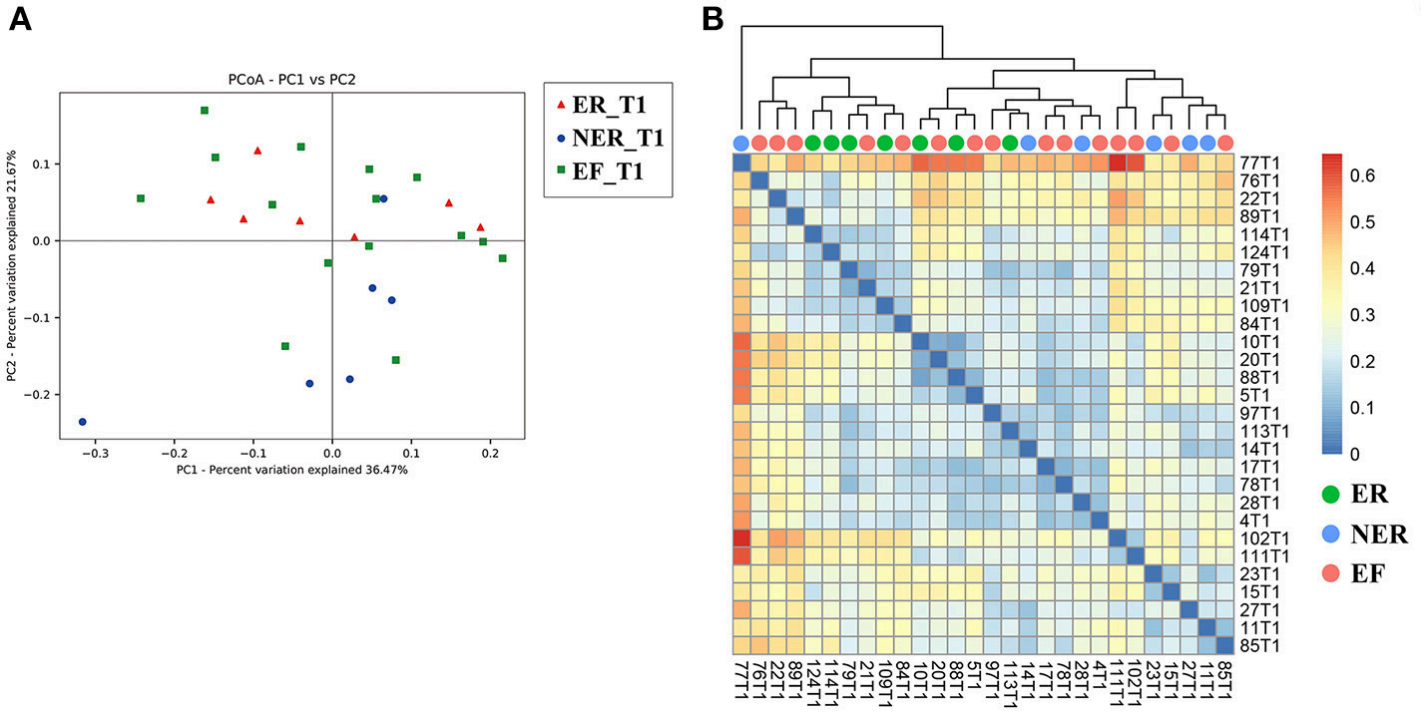
**(A)** Principal coordinates analysis (PCoA) based on weighted UniFrac distance of community structure at baseline. Each symbol represents one sample: red for the ER group (*n* = 7); blue for the NER group (*n* = 6); green for the EF group (*n* = 15). **(B)** Correlation-Heatmap based on weighted UniFrac distance of the three groups at baseline. Trees were clustered based on weighted UniFrac distance: green for the ER group (*n* = 7); blue for the NER group (*n* = 6); tangerine for the EF group (*n* = 15).

To investigate if the differences in the structure of salivary microbial community were associated with the recurrence of ECC, samples of the three groups at baseline were clustered via PCoA based on weighted UniFrac distance (Figure 3A). The results showed that the ER and NER groups both had a considerable overlap with the EF group on T1 (Anosim method, *P* = 0.955 for the ER vs. EF group, and *P* = 0.288 for the NER vs. EF group, respectively), whilst the ER group had statistically significant separation with the NER group on T1 (Anosim method, *P* = 0.039 for the ER vs. NER group), indicating that the microbial community structure exhibited significant differences between the ER and NER groups at baseline. We also assessed the predictive potentiality of this structure for ECC recurrence at baseline based on weighted UniFrac distance (Figure 3B). The clustering tree revealed that we could distinguish most of the samples from ER and NER group by their microbial community structures at baseline, which provided evidence for using salivary microbial community structure to predict the recurrence of ECC at an early phase.

### Potential Biomarkers Discovered by Comparison of Relative Abundance at the Genus Level

**Figure 4:**
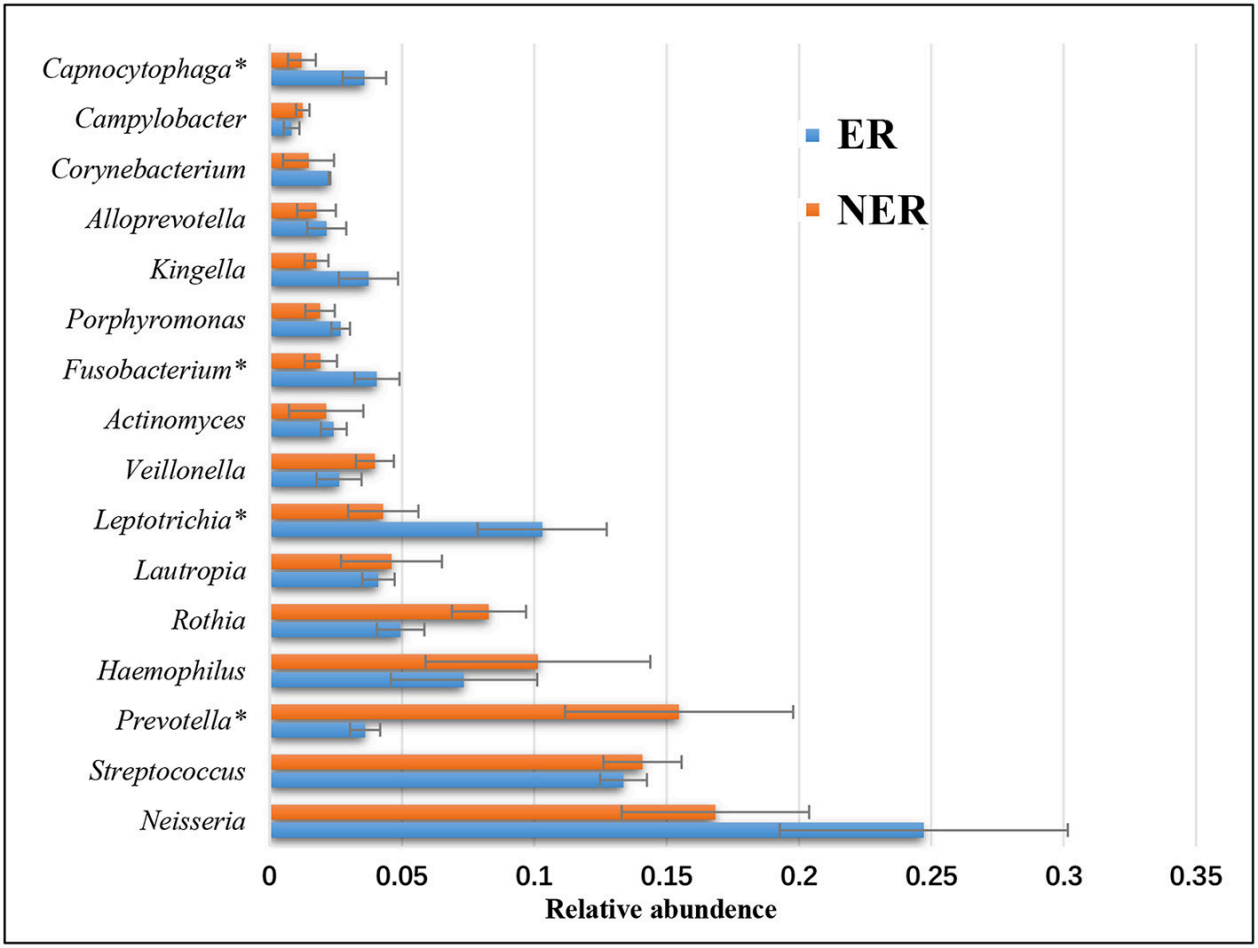
Comparison of relative abundance of bacteria by genus between the ER and NER groups at baseline. *P < 0.05.

The relative abundance of genera between the ER and NER groups were compared, and any taxa with a median relative abundance below 1% were excluded. The relative abundance of predominant bacteria by genus was shown in Figure 4. At the genus level, *Neisseria, Streptococcus, Prevotella, Haemophilus, Rothia, Lautropia, Leptotrichia, Veillonella, Actinomyces, Fusobacterium, Porphyromonas, Kingella, Alloprevotella, Corynebacterium, Campylobacter*, and *Capnocytophaga* dominated the communities in the ER and NER groups. A group of 4 bacterial genera, including *Fusobacterium, Prevotella, Leptotrichia*, and *Capnocytophaga*, showed statistically significant differences in abundance between the ER and NER groups using the metastats method (*P* = 0.049, *P* = 0.025, *P* = 0.020, and *P* = 0.035, respectively). In the saliva sample from children with history of ECC treatment, higher abundance of *Fusobacterium, Leptotrichia*, and *Capnocytophaga*, and lower abundance of *Prevotella*, would be potential indicators for the recurrence of ECC.

### Predictive Analysis of the Recurrence of ECC Using Detected Genera

**Figure 5:**
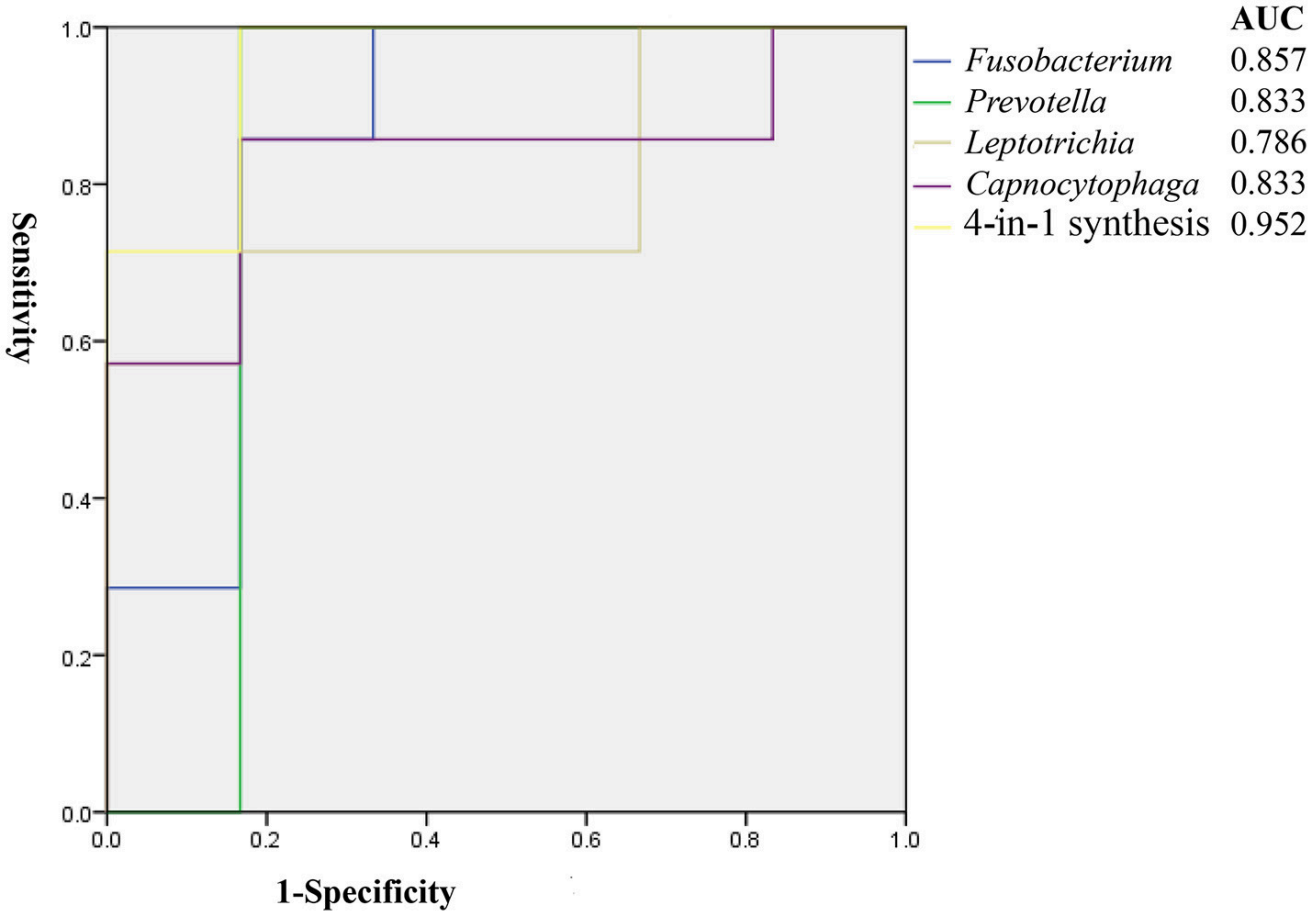
Receiver-operating characteristic (ROC) analysis of ECC recurrence using detected genera assessed by the area under the curve (AUC).

We conducted the receiver-operating characteristic (ROC) analysis for the recurrence of ECC using the four genera detected by comparisons of relative abundance of predominant bacteria at the genus level between the ER and NER groups. The results were shown in Figure 5, and the discriminatory capability was evaluated using Area Under the Curve (AUC). The analysis of each genus was based on the relative abundance in each sample, and the formula of our 4-in-1 synthesis was set as the summary of relative abundance of *Fusobacterium, Leptotrichia*, and *Capnocytophaga* minus that of *Prevotella*. Finally, we got the AUC of *Fusobacterium, Prevotella, Leptotrichia, Capnocytophaga* and 4-in-1 synthesis were 0.857, 0.833, 0.786, 0.833, and 0.952, respectively. This indicated that the predictive method for the recurrence of ECC based on one of the four genera above was able to show satisfactory accuracy, and the method based on their combination could be even better.

## Discussion

ECC is a critical public health concern and the poor clinical outcomes owing to high incidence of recurrence after ECC treatment exacerbates its challenge (Almeida et al., 2000). Technical development for risk assessment and early prediction of ECC recurrence has become both significant and urgent (Selwitz et al., 2007). A number of previous studies have reported traditional predictors of caries relapse after dental treatment, but some of them remain controversial (Berkowitz et al., 2011; Amin et al., 2015) with apparent limitations in the existing methods (Tellez et al., 2013). Novel approaches based on best evidence are needed to improve the prevention of recurrence of this disease.

With the rise of the ecological plaque hypothesis for dental caries, study of the composition and characteristics of oral microbiome from an overall perspective has become the focus of research. According to recent advances in understanding the role of the oral microbiome in health and disease, the development of oral microbiota in preschool children appears to be driven in part by changes in the relative abundance of taxa rather than the acquisition/loss of certain taxa (Yang et al., 2012; Jiang et al., 2014; Zaura et al., 2014). This suggests that measures on features and dynamics of microbiome could be appropriate to define probable risk, onset, progression and recurrence of ECC (Hemadi et al., 2017). As the collection of saliva is quick, non-invasive, and easy to perform in home care, methods based on salivary microbiota can potentially serve as a targeted, sensitive, and patient-friendly measure for risk assessment and detection of ECC (Kaczor-Urbanowicz et al., 2017). In the present study, in order to ascertain whether the microbial composition of saliva after treatment could help us predict which children had the highest risk for the recurrence of ECC, we developed a method which had predictive potentiality for ECC recurrence based on salivary microbiota. The identification of children with high risk of relapse at an early post-therapy stage would be helpful for taking appropriate preventive measures before recurrent lesions occur.

In the sequencing analysis, the rarefaction curves (Figure S1B) and the specaccum curve (Figure S1C) confirmed that sequencing depth of all the samples and the sample size was reasonable. The comparison of Shannon microbial diversity indices (Figures 1C,D) showed no statistically significant differences in both cross-sectional and longitudinal comparisons. Similar to our observations, some previous studies had also shown no significant difference in the α-diversity of those with and without caries (Griffen et al., 2012; Xu et al., 2014; Johansson et al., 2016; Zhou et al., 2017), however some other studies had shown that increased α-diversity favored healthy status (Gross et al., 2010; Belstrom et al., 2014; Xiao et al., 2016). The contradiction amongst the results was brought by the sampling methods, analysis methodologies, or ethnic groups. Therefore, further studies are needed to verify this issue. In the present study, 13 known bacterial phyla and 85 genera derived from 3,650,544 sequences were detected from 84 samples, which suggested that this sequencing analysis could provide the details of the oral microbiota. The level of microbiome diversity in the present study was consistent with sequencing studies in other population with similar conditions (Jiang et al., 2014, 2016). Besides, the results of β-diversity comparison in our study (Figure 2) indicated that saliva from children with and without ECC exhibited similar microbial community structure.

PCoA (Figure 3A) and heatmap plots (Figure 3B) demonstrated that microbial community structure could distinguish the ER and NER groups at baseline. As expected, the saliva microbiome features may serve as a microbial indicator to define susceptibility of ECC recurrence. In the specific comparison between the ER and the NER group at baseline, a group of f bacterial genera, including *Fusobacterium, Prevotella, Leptotrichia*, and *Capnocytophaga*, showed statistical differences in abundance. Based on their tendencies between different time points, it was inferred that higher abundance of *Fusobacterium, Leptotrichia*, and *Capnocytophaga*, as well as lower abundance of *Prevotella*, were potential indicators of the recurrence of ECC. In previous studies, *Fusobacterium, Leptotrichia*, and *Capnocytophaga* were also reported to be enriched under the status of caries occurrence (Kanasi et al., 2010; Ling et al., 2010; Jiang et al., 2013). But intriguingly, enriched *Prevotella* had been reported to be associated with ECC (Palmer et al., 2010; Tanner et al., 2011), however in our present study the lower abundance of *Prevotella* favored ECC recurrence, indicating that oral microbiome might play a different role in the courses of onset and relapse of caries. Furthermore, studies showed that *Prevotella* species were not similarly distributed between caries and caries-free population, whilst several species of *Prevotella* (*Prevotella* spp., *Prevotella shahii, Prevotella pallens*) presented at a much higher level in the caries-free group than the caries group (Yang et al., 2012; Jiang et al., 2016; Gomez et al., 2017). Therefore, species-level and potentially even strain-level resolution might be important for the issue. *Prevotella* did not fit the classical characteristics of cariogenic organisms as acid-tolerant or even acid-sensitive (Simon-Soro and Mira, 2015). Thus, whether *Prevotella* play an active role or are just bystanders in cariogenesis still needed to be further explored.

Receiver-operating characteristic (ROC) analysis was proven to be effective in a previous study for establishment of the predictive model (Teng et al., 2015). In the present study, the AUC of *Fusobacterium, Prevotella, Leptotrichia, Capnocytophaga* and their combination for prediction for ECC recurrence were 0.857, 0.833, 0.786, 0.833, and 0.952, respectively. These values were considerably higher when compared with a predictive study on the onset of ECC (Teng et al., 2015). Further studies on the probable mechanism of the pathogenesis and progression of ECC recurrence might yield better understanding of the nature.

Some limitations should be considered for the present research. First, only 28 children were recruited in this study, which sample size might not be so sufficient for a predictive study to move further to conduct subgroup analysis with regards to the status of ECC recurrence. Second, the sequencing technology used in this study was based on Illumina MiSeq PE250 platform, which had difficulty to provide effective resolution below the bacterial genus level. In our present study, the OTUs were not aligned well at species level (the unidentified OTU percent at species level ranged from 62.2 to 89.5%), thus these species-level data were not provided to avoid potential bias. For in-depth understanding the etiology of caries, it is expected that further studies on oral microbiota could utilize novel techniques to perform analyses at the species level. Third, dental caries is a complicated multifactor disease, which is caused not only by microbial infection, but also associated with many other factors from the host and environment. Although these factors were not fully considered in this study, we were recruiting children from the same kindergarten who had the same regular diets and oral health instructions, and they shared similar living environments. This could considerably reduce the probable impacts resulted by some other background factors. Fourth, oral microbiome was reported to be influenced by a number of hereditary or environmental factors (Li et al., 2014; Gomez et al., 2017; Gao et al., 2018), but the present study only covered the population of Chinese preschool children in Beijing, which should be interpreted with caution when considering other ethnic groups. Fifth, the pathogenic mechanism associated with the verified microbial indicators of ECC recurrence is still unrevealed. Further studies are needed to explore the specific pathogenic mechanism and signal transduction pathways in the recurrence of ECC.

In addition, for now there is no study using advanced technology (high-throughput approach and mass spectrometry) to investigate the profile of saliva microbiome and proteome at the same time for prediction of dental caries. As previously reported, physiological state of the human body could be detected by monitoring changes in the composition of saliva (Banderas-Tarabay et al., 2002; Van Nieuw Amerongen et al., 2004). Proteins are also the principal ingredient in saliva (Chiappin et al., 2007). Proteins and peptides contained in saliva are important to maintain oral health and homeostasis, since the severity and frequency of oral disease are often associated with the qualitative and quantitative changes of salivary proteome/peptideome (Dawes et al., 2015). Caries risk assessment could be conducted by investigating the bacterial abundance, protein identity and concentration. Further work with both salivary microbiome and proteome/peptideome considered is expectable to fill this gap and contribute to our knowledge on the recurrence of ECC. Moreover, longitudinal monitoring and surveillance could help understand how salivary microbiome and proteome/peptideome changed in ECC recurrence after management and interventions of the disease. In the future, once the systematic biomarkers for the ECC recurrence were verified, the findings could push forward the translation of rapid chair-side test for clinical practice or self-monitoring for home care.

In conclusion, salivary microbiome exhibited potentiality for predicting the recurrence of ECC at an early phase. *Fusobacterium, Prevotella, Leptotrichia*, and *Capnocytophaga* were associated to the pathogenesis and progression of ECC recurrence. The method using relative abundance of these four bacterial genera and their combination to predict the recurrence of ECC showed satisfactory accuracy. These findings might facilitate more effective strategy to be taken in the management of the recurrence of ECC.

## Data Availability

The raw sequencing data of this study are available in the NCBI Sequence Read Archive with accession number SRP162752.

## Author Contributions

CZ, CY, SA, XYS, and SZ contributed to the concept, design, data acquisition, analysis, and interpretation and drafted and critically revised the manuscript. XRS and FC drafted and critically revised the manuscript. All of the authors have read and approved the final manuscript.

### Conflict of Interest Statement

The authors declare that the research was conducted in the absence of any commercial or financial relationships that could be construed as a potential conflict of interest.

## Supplementary Material

Table S1 The checklist for STROBE guidelines.

Table S2 The alpha-diversity indices in each group.

Figure S1 **(A)** OTU distribution between the three groups for each time point. **(B)** The rarefaction curves for each time point. **(C)** The specaccum curves for each time point.

Figure S2 Relative abundance of taxa from phylum to genus level in each sample at T1.

Figure S3 Relative abundance of taxa from phylum to genus level in each sample at T2.

Figure S4 Relative abundance of taxa from phylum to genus level in each sample at T3.
