# Supplementary material for: The Predictive Potentiality of Salivary Microbiome for the Recurrence of Early Childhood Caries
Source: Front Cell Infect Microbiol. 2018 Dec 14;8:423. doi: 10.3389/fcimb.2018.00423 (PMC6302014; doi:10.3389/fcimb.2018.00423)
Supplement: Table S2 — The alpha-diversity indices in each group. [file Table_2.docx]

| TABLE S2. The alpha-diversity indices in each group. | | | | |
| --- | --- | --- | --- | --- |
| Groups | Chao 1 index (Mean±SD) | Observed species index (Mean±SD) | PD_whole_tree index (Mean±SD) | Shannon index (Mean±SD) |
| ER_T1 | 149.35±13.167 | 137.30±14.769 | 11.97±1.065 | 4.59±0.652 |
| ER_T2 | 152.78±12.358 | 140.53±12.162 | 12.51±1.016 | 4.17±0.537 |
| ER_T3 | 146.45±10.228 | 134.43±11.046 | 12.18±1.043 | 4.08±0.506 |
| NER_T1 | 131.02±13.790 | 119.33±13.239 | 11.46±0.926 | 4.46±0.301 |
| NER_T2 | 136.94±9.118 | 127.07±11.637 | 11.75±0.935 | 4.74±0.421 |
| NER_T3 | 121.11±22.515 | 110.58±24.391 | 10.28±1.898 | 4.24±0.603 |
| EF_T1 | 137.54±18.301 | 122.47±19.152 | 11.37±1.719 | 4.36±0.649 |
| EF_T2 | 141.09±18.159 | 126.14±20.632 | 11.79±1.653 | 4.52±0.571 |
| EF_T3 | 150.82±13.257 | 136.85±14.219 | 12.31±1.081 | 4.61±0.572 |
