# Supplementary figures and images for: The Predictive Potentiality of Salivary Microbiome for the Recurrence of Early Childhood Caries
Source: Front Cell Infect Microbiol. 2018 Dec 14;8:423. doi: 10.3389/fcimb.2018.00423 (PMC6302014; doi:10.3389/fcimb.2018.00423)

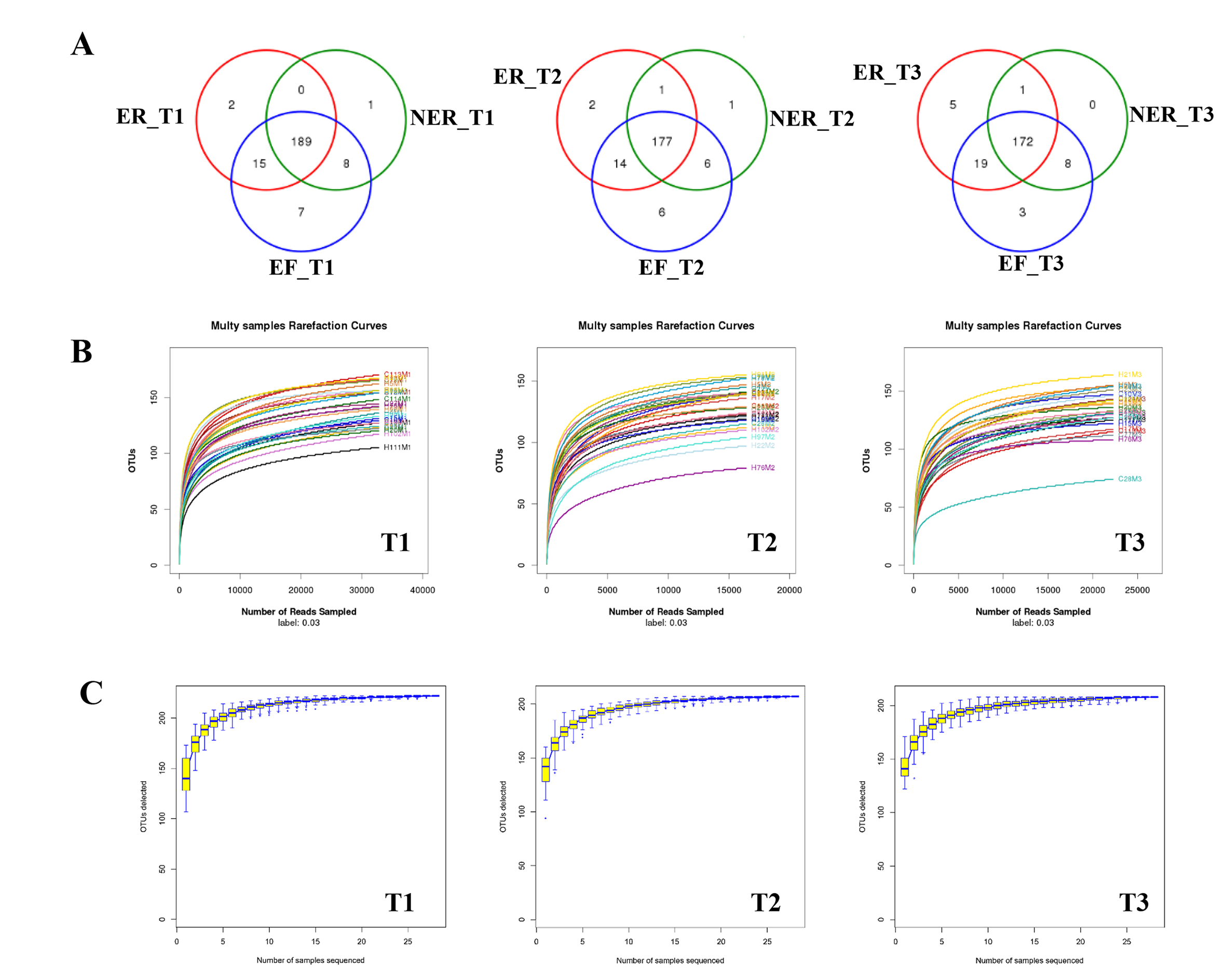

Supplement: Figure S1 — (A) OTU distribution between the three groups for each time point. (B) The rarefaction curves for each time point. (C) The specaccum curves for each time point. [file Image_1.TIF]

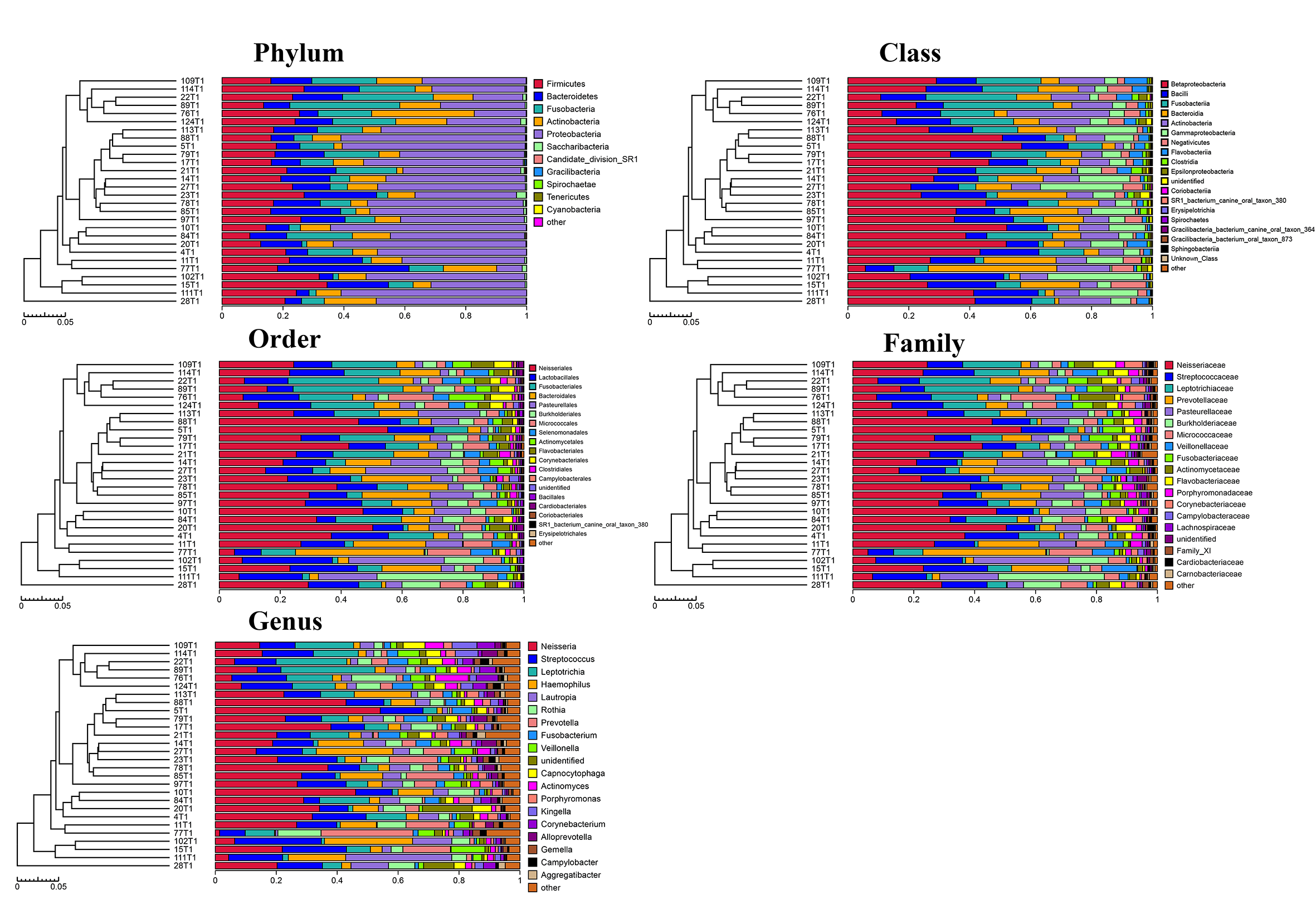

Supplement: Figure S2 — Relative abundance of taxa from phylum to genus level in each sample at T1. [file Image_2.TIF]

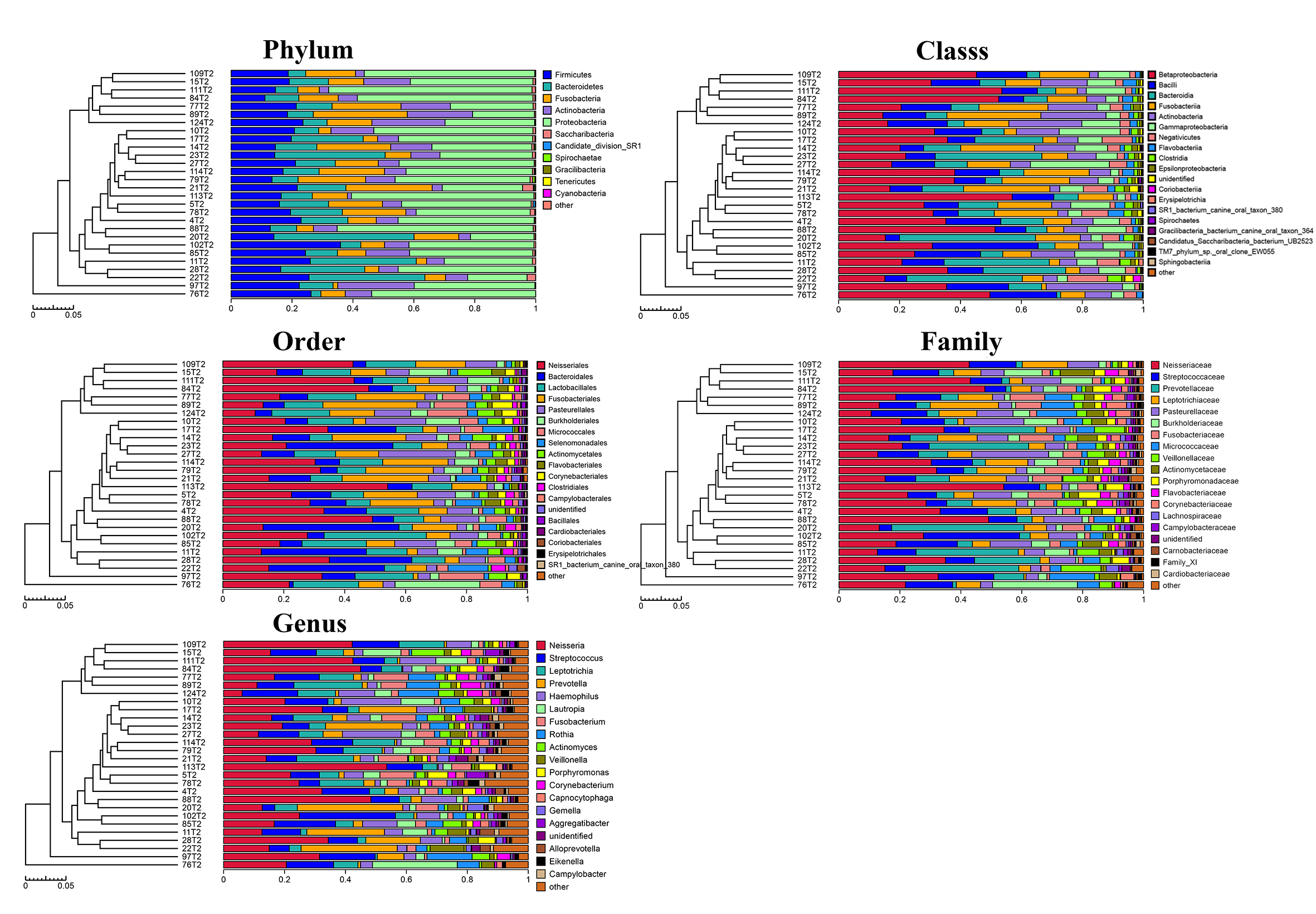

Supplement: Figure S3 — Relative abundance of taxa from phylum to genus level in each sample at T2. [file Image_3.TIF]

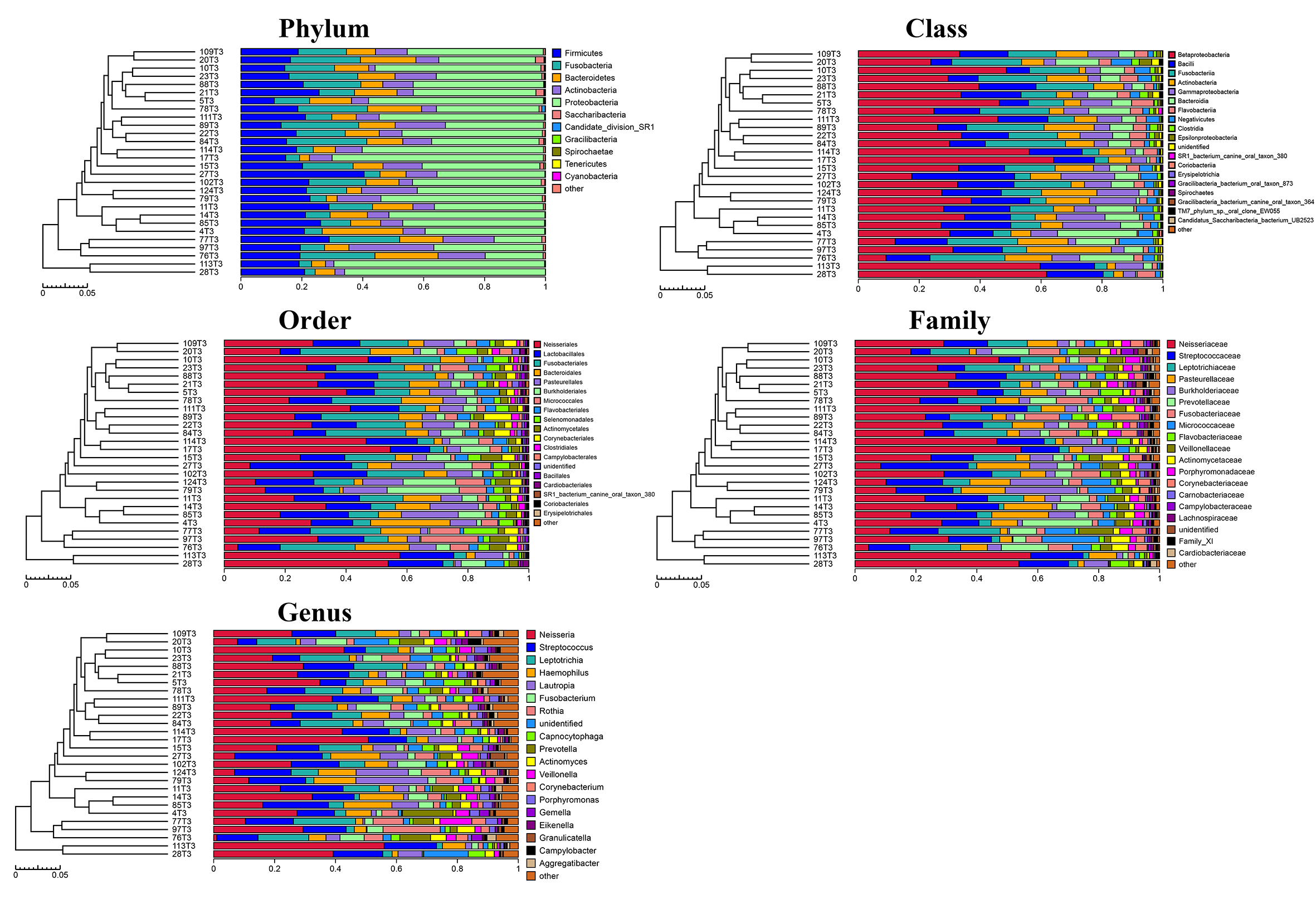

Supplement: Figure S4 — Relative abundance of taxa from phylum to genus level in each sample at T3. [file Image_4.TIF]
